# Do COVID-19 responses associate with prior hazard experiences? An investigation among flood-vulnerable subsidized housing residents with digital vulnerability

**DOI:** 10.3389/fpubh.2025.1569090

**Published:** 2025-05-30

**Authors:** Seungbeom Kang, Dabin Lee, Jooyoung Kim, Jiahn Lee, Jongho Won, Yan Wang

**Affiliations:** ^1^Department of Urban Planning and Engineering, Yonsei University, Seoul, Republic of Korea; ^2^Department of Urban Design and Planning, Hongik University, Seoul, Republic of Korea; ^3^Department of Urban and Regional Planning, University of Florida, Gainesville, FL, United States

**Keywords:** digital divide, COVID-19, disaster preparedness, digital skills, logistic regression

## Abstract

In light of the threat posed by the COVID-19 pandemic and the accompanying public health risks, understanding the mechanisms through which individuals discern accurate information about COVID-19 and adopt appropriate preventive behaviors has become an important research subject. However, few studies have directly examined the associations between the digital divide, previous experience of non-pandemic disasters, and preventive behaviors in response to COVID-19. This study focuses on two elements that may affect individuals’ responses to COVID-19: (1) digital capabilities and (2) prior experience of and preparedness for flood risk. This study analyzed survey data collected from 200 households residing in flood-vulnerable subsidized housing in Florida, USA. The findings demonstrate that proficiency in Internet search skills is strongly and positively associated with information-seeking and preventive behaviors against COVID-19, while social media usage skills did not produce the same association. Moreover, the variables that indicate experience with and risk mitigation for flood hazards are significantly associated with the diversity of channels used to search for COVID-19 risk information, information-seeking behaviors, and preventive actions. These results suggest that improving preparedness for non-pandemic events may also enable individuals to be better prepared for future pandemics. The findings provide several action-oriented policy implications for reducing the multiple forms of vulnerability to which residents of subsidized housing are exposed.

## 1 Introduction

The outbreak of coronavirus disease 2019 (COVID-19) in December 2019 rapidly emerged as one of the most significant threats to urban life globally and human existence in recent history. In response to its unprecedented global transmission, the World Health Organization (WHO) declared COVID-19 a pandemic in March 2020. As of September 30, 2022, over 609 million confirmed cases and over 6.5 million deaths had been reported, including approximately 95 million cases and one million deaths recorded in the United States alone^1^.

The global spread of COVID-19 has prompted a wide range of compliance behaviors at both the individual and national levels. At the individual level, preventive measures include wearing face masks, staying at home, avoiding public or crowded places, avoiding travel, avoiding contact with high-risk individuals, washing or sanitizing hands, and engaging in social distancing (1). At the national level, the federal government implemented various actions, including quarantine and social distancing protocols, closure of non-essential businesses and schools, stay-at-home orders, and public guidelines on preventive practices (1).

Given this context, understanding how individuals perceive COVID-19 risks and identifying factors that influence their adoption of preventive behaviors becomes crucial. Previous studies have identified a wide range of factors shaping preventive behaviors, spanning from national policies to individual characteristics. These include mandatory administrative and legislative actions (e.g., stay-home restrictions), (in)consistent guidelines across different nations and health organizations, differences in attitudes and degrees of autonomous motivation, discrimination against individuals who wear face masks in public areas, subjective norms, and perceived behavioral control (2, 3). Moreover, adherence to preventive perceptions and behaviors also varies according to demographic and socioeconomic characteristics—such as sex, age, presence of older relatives, educational attainment, urban residence, presence of family members with pre-existing health conditions, political ideology, and the availability of social support (1, 4–8).

However, it has been consistently challenging to encourage individuals to adopt the appropriate preventative behaviors. The literature on disaster mitigation has emphasized the importance of adequate communication between governmental entities and individuals (9). The literature specifically focuses on the ability of individuals to acquire reliable information, as substantial information regarding disaster risks is disseminated via digital media, which frequently includes misinformation (10, 11). Despite these potential theoretical relationships, few studies have directly examined the associations among the digital divide, information-seeking, and preventive behaviors in response to the COVID-19 pandemic.

### 1.1 Research purposes and questions

This study aimed to explore the intersections between multidimensional digital divides, flooding risk preparation as a proxy of prior disaster experiences, and information–seeking and preventive behaviors in response to the COVID-19 crisis by examining exploring two under-examined research questions: (1) Are digital skills–including internet and social media proficiency–positively associated with information-seeking and preventive behaviors in response to COVID-19? (2) Is prior experience with and preparedness for flood risks positively related to information-seeking and preventive behaviors in response to COVID-19?

### 1.2 Target population: flood-vulnerable subsidized housing residents in Florida

We particularly focus on examining one vulnerable subgroup that was placed in multi-hazard situations: subsidized housing residents who reside in flood-prone areas in Florida USA; hereafter, referred to as flood-vulnerable subsidized housing (FVSH) residents. Exploring this group provides an opportunity to investigate how marginalized groups’ preventive behaviors during COVID-19 are shaped by their information–seeking skills and prior disaster experience—directly addressing the two primary contributions of this study. Subsidized housing residents tend to be overrepresented in multi-hazard situations, such as sea-level rise and coastal floods (12, 13). Similarly, FVSH residents who are particularly vulnerable to flooding due to their limited resources also often have characteristics—including racial minorities, people who cannot work remotely, and older adults—that are likely to face greater COVID-19 infection risks (14–20). That is, the reliance on government subsidies limits FVSH residents’ financial flexibility and housing options for addressing their environmental vulnerability to COVID-19. Moreover, their constrained income also contributes to multidimensional multidimensional DD, particularly among individuals with partial but limited digital access and skills. These residents are not entirely disconnected, but often fall into a digitally marginal group whose online engagement is constrained in meaningful ways. This population represents an important but underexplored segment of digital vulnerability. Several surveys have already shown that subsidized housing residents were likely to have lower high-speed internet access compared to other populations and frequently experience under-connectivity (21, 22). Therefore, focusing on FVSH residents provides a great opportunity to address a wide range of topics: multidimensional digital divides, prior disaster experience, and COVID-19 preventive responses.

### 1.3 Previous studies and research gaps

To clarify the theoretical underpinnings and scholarly contributions of this study, we review two key strands of literature that inform the research questions. First, this study integrates the concept of the digital divide (DD) as the WHO labeled the plethora of COVID-19-related information available as an “infodemic” (23)–a phenomenon highlighting challenges posed by misinformation that undermines public health responses and fosters confusion. The DD was originally proposed in the 1990s to describe uneven adoption or penetration of the Internet and other Information and Communication Technologies (ICTs) among various sociodemographic groups. As ICTs have advanced and the usage of smart devices has grown exponentially, DD evolved beyond a simple accessibility gap by encompassing the following: (1) the skills and abilities required to use ICT (i.e., the second-level DD) (24) or (2) the skills and abilities required to enable practical use of the Internet (i.e., the third-level DD) (25). These expanded dimensions of DD intensified disparities among demographic and socioeconomic groups, consequently impacting their capacity to effectively utilize digital public resources and respond appropriately to emergencies (10, 25–29). For example, older adults, lower-income and less-educated households, and those with restricted Internet access are more prone to experience social isolation and increased vulnerability during pandemics (30–32). Consequently, the widening DD may exacerbate disparities in individuals’ responses to COVID-19, particularly during stay-at-home orders that increase dependence on digital sources for information and social connection. Despite these potential roles of DD in shaping various responses to COVID-19, few studies have directly examined the associations among the digital divide, information-seeking, and preventive behaviors in response to the COVID-19 pandemic.

Second, this study also explores the relationship between prior experience of flooding—non-pandemic disaster—and preventive behaviors in response to COVID-19. Research indicates that exposure to multiple disaster risks over time is not unusual as approximately one-third of natural disaster survivors experienced another form of disaster (33, 34). While repeated exposure to disasters can strain individuals’ adaptive resources, and affect survivors’ sense of control, predictability, safety, and trust (35–37), previous disaster experiences can also yield positive outcomes—such as including increased resilience, self-esteem, and self-control (36, 38). The conservation of resource stress model (39) suggests that individuals accumulate resources from previous disaster experiences to better cope with future disaster threats. Similarly, the warning and response model (40) proposes that situational factors (e.g., risk communication), personal characteristics (e.g., age, education, disaster experience), and social contexts (e.g., family context) influence perceptions of threat and protective actions. These perspectives collectively suggest a theoretical relationship between prior disaster experience, information-seeking, and preventive behaviors during subsequent disasters, such as COVID-19. However, to date, little evidence exists regarding responses to COVID-19 among individuals exposed to other non-pandemic risks (Table 1).

**Table 1:** Descriptive statistics of dependent and key independent variables.

Variable	Obs	Percentage (%)
Accessibility to internet	200	
No subscription for any internet service		4.0
Through a high-speed internet subscription (e.g., Comcast, Cox, AT&T)		47.0
Through a smartphone data plan (e.g., Sprint, Verizon, AT&T, T-Mobile)		14.0
Dual subscriptions (high-speed internet and smartphone data plan subscriptions)		35.0
Proficiencies in internet searching skills	192	
Low level of internet searching skills		26.0
Medium level of internet searching skills		20.8
High level of internet searching skills		53.1
Proficiencies in internet searching skill	200	
Low level of social media usage skill		26.0
Medium level of social media usage skill		27.5
High level of social media usage skill		46.5
Sources of acquiring COVID-19-related information	200	
Relying on mass media		3.0
Relying on online information		10.5
Relying on governments or experts		5.5
Relying on personal networks		4.5
Relying on more than two sources		76.5
Engagement in preventive behaviors against COVID-19	198	
Passive preventive behaviors		36.9
Selective preventive behaviors		31.3
Active preventive behaviors		31.8
COVID-19-related information-seeking behaviors	198	
Information non-seekers		30.8
Passive information seekers		43.4
Active information seekers		25.8
Degree in disaster mitigation	200	
Taking minimal preventive behaviors in disaster mitigation		36.5
Taking passive preventive behaviors in disaster mitigation		24.5
Taking active preventive behaviors in disaster mitigation		39.0
Previous experience with flooding	197	
Experienced flooding		36.0
Never experienced any flooding		64.0

## 2 Materials and methods

This study aimed to explore the intersections between multidimensional digital divides, flooding risk preparation, and information–seeking and preventive behaviors in response to the COVID-19 crisis by FVSH residents in Florida, USA. Based on a review of the existing literature, three sets of hypotheses were proposed.

First, building on prior research that highlights the influence of Internet access and digital skills on individuals’ preventive and information-seeking behaviors during the COVID-19 pandemic, we examined whether FVSH residents experiencing lower levels of digital divide (DD) would be more likely to seek diverse COVID-19-related health information and engage in preventive actions (H1). Specifically, we hypothesized that residents with higher proficiency in using the Internet or social media would acquire more diverse information (H1a/b) and engage more actively in prevention behaviors (H1c/d).

Second, drawing on literature concerning cumulative or interacting disaster experiences, we tested whether prior experience with flooding and a high degree of flood preparedness would promote proactive information-seeking and preventive behaviors in response to COVID-19 (H2). We posited that residents who had previously experienced flooding or were better prepared would be more likely to acquire diverse information (H2a/b) and take preventive actions (H2c/d).

Third, we considered the possibility that prior flooding experience and preparedness may mediate the relationship between digital skills and COVID-19-related behaviors. Thus, we hypothesized that after controlling for flooding variables, digital skills would no longer be significantly associated with information-seeking or preventive behaviors (H3a–d).

To test these hypotheses, we conducted a targeted survey of FVSH residents and analyzed their responses using a range of descriptive and multivariate statistical techniques. The survey was conducted using 200 FVSH residents aged over 18 years who were household heads as the sample group. The questionnaire was designed to elicit information pertinent to this study, such as Internet service access, Internet search proficiency, social media skill proficiency, previous experience with flooding, flood preparedness activities, sources of acquiring COVID-19-related information, engagement in COVID-19-preventive behaviors, and COVID-19-related information-seeking behaviors. For survey distributions, we sorted 798 ZIP Code Tabulation Areas (ZCTA) zones that (1) contained more than 25% subsidized housing units and (2) were located in 100-year or 500-year floodplains. We elected to focus on the Florida region because it has many areas prone to hurricanes and flooding, and many racially and socioeconomically diverse neighborhoods. Potential respondents were recruited through the survey company Qualtrics to fill a panel with a set of nested quotas. We utilized the stratified cluster sampling method, which allowed us to stratify the recruited participants based on local population structure, providing a more representative sample of flood-vulnerable households in the study region.^2^ Potential respondents received an email invitation containing a secure URL through which to access the survey and review its intended purpose. 200 responses were obtained from subsidized housing residents out of 1,312 gathered responses to the screening question, “Do you live in subsidized housing?” All data collection procedures were approved by the Institutional Review Board (IRB) of the University of Florida.

Despite the inherent limitation of online recruitment, it is important to emphasize that our study intentionally targeted a digitally marginal population—those who have some internet access but lack the skills or confidence to use it effectively in risk scenarios. Given the constraints of the COVID-19 pandemic, online recruitment was the most feasible method for reaching this population in a timely and safe manner. The multi-step screening process further allowed us to selectively capture individuals living in both subsidized housing and high-risk flood zones, aligning with the study’s objective of examining vulnerability across environmental and digital dimensions. Thus, while our method did not encompass individuals entirely disconnected from digital platforms, it remains methodologically coherent with our research focus.

The survey data were collected in March 2021, a period when the state of Florida officially urged citizens to maintain appropriate social distancing and sanitation protocols. Although some public health measures remained in place, such as encouraging mask use and limiting large gatherings, the state had already lifted many mandatory restrictions by late 2020. At that time, Governor Ron DeSantis emphasized individual responsibility over government mandates, a stance that diverged from federal guidelines issued by the Centers for Disease Control and Prevention (CDC). Meanwhile, COVID-19 vaccinations had only recently begun to be administered, primarily to older adults and high-risk groups. According to the CDC, less than 10% of Florida’s total population was fully vaccinated by the end of March 2021. These contextual factors are important when interpreting public attitudes toward official health information, as the accuracy and consistency of government guidance during this phase of the pandemic were variable and, at times, politically contested.

Table 2 presents descriptive statistics of the survey respondents’ key characteristics. Two-thirds of respondents (66%) were between the ages of 10 and 20 years. More than half were unmarried (55%), and there were more female (57%) than male (41%) respondents. Approximately 35% of the respondents did not have a college degree. Half of the respondents had a total household income of $30,000 or more, while the remaining respondents had less. More than half of the respondents were white or Caucasian, followed by black/African-American (32%) with the remaining respondents being of other races. Approximately 48% of respondents had full-time jobs, 23% were employed part-time, and the remaining 29% were made up of students, retirees, and other unemployed individuals. All of these demographic, economic, and social characteristics were included in the analytic models as control variables.

**Table 2:** Descriptive statistics of control variables.

Variable	Obs	Percentage (%)
Age	198	
10s-20s		66.2
30s		17.2
40s		7.6
50s		2.5
60+		6.6
Race/Ethnicity	197	
White		39.1
Black or African-American		23.3
Hispanic, Latino, Spanish		28.4
Asian or Asian American		1.0
Native Hawaiian or Pacific Islander		0.5
Some other race		7.6
Gender	200	
Male		41.0
Female		56.5
Others		2.5
Married	200	
Single, never married		55.0
Married or domestic partnership		39.0
Widowed, Divorced, Separated		6.0
Education	200	
Under or equivalent to level of high school		29.5
Below college		35.0
Bachelor’s degree		22.0
Above bachelor		13.5
Employment type	200	
Full time		52.0
Part-time		21.0
Unemployed		27.0
Household income	200	
Income ($0- $14,999)		17.5
Income ($15,000–$34,999)		30.0
Income ($35,000-more)		52.5

### 2.1 Measures and descriptive statistics

In addition to socioeconomic and demographic characteristics, Internet accessibility, Internet proficiency and social media skills, acquisition of COVID-19-related information, engagement in preventive behaviors, channels for receiving risk information, experience with flooding hazards, and disaster mitigation levels were all assessed.

Respondents’ information-seeking behaviors in response to COVID-19 were evaluated by asking, “What health information about COVID-19 have you received?” Respondents were allowed to choose multiple responses. The following options were provided in the questionnaires: (a) the rate of infection or death in a respondent’s county or state, (b) the scientific facts of the pandemic, (c) preventive measures, and (d) social support, and others. Respondents were classified into three groups based on the number of selected responses: information non-seekers, passive information seekers, and active information seekers (Table 2). Approximately 43% of the respondents passively sought COVID-19-related information, while the remaining respondents were either information non-seekers (30.8%) or active information seekers (25.8%).

We measured the degree of engagement in preventive behaviors, which reflects the specific actions taken by FVSH residents in response to COVID-19. Based on responses to the question, “How have you engaged in preventive behaviors against the coronavirus?” We categorized respondents into three mutually exclusive groups: (1) those who engaged in preventive behaviors passively (i.e., no preventive or social distancing actions), (2) those who engaged in preventive behaviors selectively (i.e., either preventive or social distancing actions), and (3) those who actively engaged in preventive behaviors (i.e., both preventive and social distancing actions). The respondents indicated proportions that were comparable for each category. Moreover, we measured specific sources of acquiring COVID-19-related information by asking, “What are the sources for you to acquire COVID-19-related information?” We confirmed that the majority of respondents (76%) acquired COVID-related information from more than one source, whereas the others utilized only a single source (e.g., mass media, online, government, and personal networks).

Given the focus of this study on the digital divide and flooding-related experiences, we constructed five independent variables that reflect Internet access, Internet search skills, social media usage skills, experience with flooding, and the degree of flooding mitigation.

Internet accessibility was measured by asking, “How do you (anyone living in your household) access the internet in your home?” We categorized the responses into four groups: (1) no subscription for any Internet service, (2) only a high-speed Internet subscription, (3) only a smartphone data plan subscription, and (4) multiple subscriptions for both high-speed Internet smartphone data plans (Table 3). 47% of the respondents accessed the Internet through high-speed Internet subscription, while 35% of the respondents used both methods to access the Internet. Approximately 4% of the sample households did not have Internet subscriptions.

**Table 3:** Descriptions of dependent variables (Information-seeking and preventive behaviors against COVID-19).

Variable group	Variable name	Explanation	Percentage (%)	Obs
**COVID-19-related information-seeking behaviors**		**Question**: *What COVID-19-related health information have you received? (Potential answers: information regarding the rate of affected people in your county or state or fatality rates, information regarding the scientific facts of the virus or pandemic, information regarding how to prevent infection by the virus, information regarding the sources and resources to give and receive social support during the pandemic, and so on)*		198
	Information non-seekers	If the sum of the responses is one, the respondent is classified as an *information non-seeker.*	30.8	
	Passive information seekers	If the sum of the responses is two or three, the respondent is classified as a *passive information seeker.*	43.4	
	Active information seekers	If the sum of the responses is four or five, the respondent is classified as *active information seekers*.	25.8	
**The degree of engagement in preventive behaviors against COVID-19**		**Question**: *How have you engaged in preventive behaviors against the coronavirus disease?*		198
	Passively engaging in preventive behaviors	If a respondent did not engage in any preventive behavior (e.g., wearing a facemask, washing hands regularly, covering mouth when coughing, cleaning touched surfaces) and social distancing (e.g., staying home, avoiding using public transportation, avoiding social gatherings) the respondent is regarded as a person who engaged in *preventive behaviors passively*.	36.9	
	Selectively engaging in preventive behaviors	If the respondent engaged in either preventive behavior or social distancing, the respondent is regarded as a person who engaged in *preventive behaviors selectively*.	31.3	
	Actively engaging in preventive behaviors	If the respondent engaged in both preventive and social distancing, the respondent is regarded as a person who engaged in *preventive behaviors actively*.	31.8	
**Sources of acquiring COVID-19-related information**		**Question**: *What are the sources for you to acquire COVID-19-related information?*		200
	Relying on mass media	Respondent acquires COVID-19-related information from mass media (e.g., news, radio).	3.0	
	Relying on online information	Respondent acquires COVID-19-related information from online information and other internet sources.	10.5	
	Relying on governments or experts	Respondent acquires COVID-19-related information from health workers directly from governments.	5.5	
	Relying on personal networks	Respondent acquires COVID-19-related information from personal networks (e.g., property managers, family, and friends, not listed)	4.5	
	Relying on more than one source	Respondent acquires information from more than one source listed above.	76.5	

Internet searching skills were measured by collecting responses on a five-point Likert scale to the following statement: “I have no difficulty to access and comprehend information that I wanted to find online.” The responses were categorized into three groups: poor, medium, and high levels of internet searching skills (Table 3). Approximately over half of the respondents indicated a high level of Internet searching skills, whereas about one-quarter of respondents indicated a low level of skills.

Proficiency in social media usage was measured by averaging the responses to five Likert-type scale sentences. The sentences pertain to messaging, posting images, sharing videos, posting context, or connecting with others using social media. The groups were restructured according to their mean Likert scores (Table 3). Approximately 46.5% of respondents reported a high degree of social media skills, whereas the groups with low or medium levels of social media skills indicate 26 and 27.5%, respectively.

Past experience with floods was measured by asking respondents whether they had experienced a flooding event (yes = 1). More than half (64%) of respondents indicated that they had never experienced flooding.

The degree of disaster mitigation, which indicates the extent to which FVSH residents prepare for disasters in advance, is measured by asking what disaster supplies they maintain in their homes. Based on the total number of selected supply items, the responses were categorized into three groups: low, medium, and high levels of disaster mitigation preparedness. Approximately 39% of respondents indicated high levels of preparedness for disaster mitigation as they had more than eight types of supply items in their homes, whereas 36% of respondents indicated poor levels of preparedness as they had fewer than four disaster items (Table 4).

**Table 4:** The descriptions of variables related to digital divide.

Category	Variable name	Explanation	Percentage (%)	Obs
**Accessibility to Internet**		**Question**: *How does your (anyone living in your household) access the internet in your home?*		200
	No subscription	Respondent has no subscription to any internet service.	4.0	
	High-speed internet subscription	Respondent accesses the internet through a high-speed internet subscription (Comcast, Cox, AT&T).	47.0	
	Smartphone data plan	Respondent accesses the internet through a smartphone data plan (e.g., Sprint, Verizon, AT&T, T-Mobile).	14.0	
	Dual subscription	Respondent accesses the internet using both a high-speed internet subscription and smartphone data plan.	35.0	
**Proficiencies in Internet searching skills** ^*^		**Sentence**: *I have no difficulty accessing and comprehending information that I wanted to find online.*		192
		If the response to the sentence above on the five-Likert type scale is one to three, the respondent is classified as having *a low level of internet searching skills*.	26.0	
		If the Likert scale is four, the respondent is classified as having *a medium level of internet searching skills*.	20.8	
		If the Likert scale is five, the respondent is classified as having *a high level of internet searching skills*.	53.1	
**Proficiencies in social media usage skills** ^*^		**Sentences**: *I know how to share information on at least one social media platform, such as Twitter, Facebook, YouTube, Reddit, etc.* *I know how to type messages through at least one social media platform.* *I know how to post images through at least one social media platform.* *I know how to share videos through at least one social media platform.* *I often post new content on my social media accounts or participate in online discussions*.		200
		If the mean of the responses to the five sentences above asking about proficiencies in using social media (e.g., sharing information, sending messages, posting images) is zero to 3.4 the respondent is classified as having *a low level of social media usage skills*.	26.0	
		If the mean is 3.5 to 4.4 the respondent is classified as having *a medium level of social media usage skills*.	27.5	
		If the mean is above 4.5, the respondent is classified as having *a high level of social media usage skills*.	46.5	

*Refers to the variables that are measured by using the five Likert-type scales from “strongly agree” to “strongly disagree” on certain statements.

### 2.2 Analytical methods

Multiple statistical methods were utilized to explore the associations between the digital divide, previous experience with and preparedness for flooding, and COVID-19 responses. Initially, we employed descriptive analytic approaches, such as chi-square, Kendall’s tau-b, and Cramér’s V tests, to explore the associations between key dependent and independent variables. We then estimated a series of logistic regression models to examine whether those associations remained significant after controlling for measurable characteristics of the sample households. Specifically, we estimated multinomial logistic models that explained information-seeking behaviors (i.e., information non-seekers, passive information seekers, and active information seekers) and the level of engagement in preventive behaviors (i.e., passively, selectively, and actively engaging in preventive behaviors). We were particularly interested in exploring the associations between the digital divide and previous experiences with and preparedness for flooding. We also estimated a series of logistic models that explained the likelihood of acquiring COVID-19-related information from a particular source. This modeling approach explores the conditions under which FVSH residents were likely to rely on a given information source during the COVID-19 pandemic.

## 3 Results

The association between engagement in preventive behaviors and acquisition of COVID-19-related information was examined using three digital divide variables (i.e., accessibility to the Internet, proficiency in Internet searching, proficiency in social media), and flooding variables (i.e., degree of disaster mitigation and experience of flooding) conducting chi-square, Kendall’s tau-b, and Cramér’s V tests for associations.

**Figure 1:**
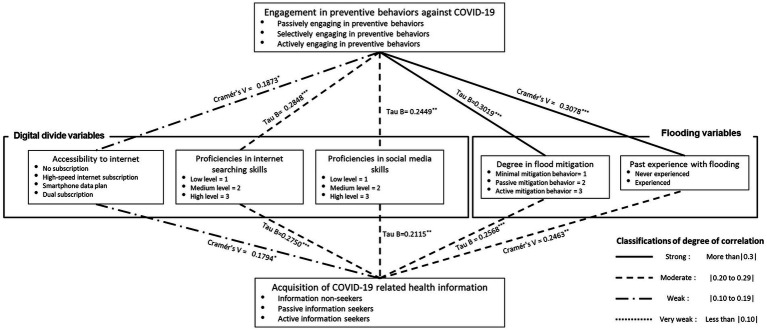
The results of descriptive analyses to examine associations between key variables. If two variables are ordinal and the cross-table is square or rectangular, we used tau B or tau C to measure the correlation of the two variables. If two variables are nominal, we used Cramér’s V to measure the correlation of the two variables. *p* + <0.1; p* < 0.05; p** < 0.01; p*** < 0.001.

Figure 1 summarizes the results obtained from examining the associations between key variables, and their statistical significance. Multiple measures of associations were incorporated, including Cramer’s V, Kendall tau, and Chi-square tests, based on the characteristics of the variables (e.g., whether a variable is ordinal or whether a cross-table is square or rectangular).

First, respondents’ digital capabilities were found to be positively associated with their COVID-19-related behaviors; specifically, as the respondents’ level of Internet access, Internet search proficiency, and social media skills increased, they became more likely to actively engage in preventive behaviors, which included both taking preventive measures (e.g., wearing a facemask, washing hands regularly) and social distancing (e.g., staying home, avoiding using public transportation). Moreover, accessibility to the Internet, Internet search proficiency, and social media skills were all found to be positively and significantly associated with actively seeking COVID-19-related health information.

Overall, flooding-related variables, including the degree of disaster mitigation and previous experience with flooding, were positively associated with actively engaging in preventive behaviors and actively seeking COVID-19-related information. Respondents who had experienced a flooding event in the past and those with a high degree of preparation for flooding tended to engage more actively in preventive behaviors and to actively seek more health information than their counterparts.

### 3.1 The results of logistic regression models

We estimated a series of logistic models to examine whether the descriptive associations identified above remained significant after controlling for other demographic (e.g., gender, race/ethnicity, age), economic (e.g., household income and employment type), and social (e.g., marital status) characteristics of sample households. First, we estimated multinomial models that explain respondents’ information-seeking behaviors (Table 5). In the models, the likelihood of being passive or active information seekers is estimated and compared with the likelihood of being information non-seekers.

**Table 5:** The descriptions of variables related to flooding.

Variable group	Variable name	Explanation	Percentage (%)	Obs
**Previous experience with flooding**		**Question**: *Have you ever personally experienced a flooding event (the building where you lived was damaged by the flood) before?*		197
	Experienced flooding	If the respondent has experienced flooding in the place they currently live or previously lived in.	36.0	
	Never experienced any flooding	The respondent has never experienced any flooding events.	64.0	
**Degree in disaster mitigation**		**Question**: *Could you tell me the disaster supplies you have in your home? (e.g., first-aid kit, flashlight, radio, water and food, extra batteries, cell phone with chargers and a backup battery, electric generator, medicine outside the current medication of you and your family members, disinfectant supplies, cloth face covering, etc.)*		200
	Low level of preparation for disaster mitigation	If the respondent has zero to three disaster supplies, the respondent is regarded as having *a low level of preparation* for disaster mitigation.	36.5	
	Medium level of preparation for disaster mitigation	If the respondent has four to seven disaster supplies, the respondent is regarded as having *a medium level of preparation* for disaster mitigation.	24.5	
	High level of preparation for disaster mitigation	If the respondent has eight to eleven disaster supplies, the respondent is regarded as having *a high level of preparation* for disaster mitigation.	39.0	

Model 1 is a reduced model that contains only digital divide-related variables. The estimated coefficients indicate that respondents who subscribed to both high-speed Internet and smartphone data plans were more likely to be passive information seekers than those who did not subscribe to any Internet service. This association was marginally significant, probably because of the limited number of respondents without internet access. For active information seekers, a high level of Internet searching skills was significantly and positively associated with being active information seekers, suggesting that the higher the level of proficiency, the higher the probability of being more active when seeking COVID-19-related health information. We found no significant evidence to support the association between social media usage skills and information-seeking behaviors.

Model 2 added a set of flooding-related variables to Model 1. The results indicate that respondents who had experienced flooding were more likely to be active information-seekers than those who had not. Moreover, compared to those with a low level of disaster mitigation – those who prepared only a few emergency items – respondents with a high degree of disaster mitigation were more likely to be active information seekers during COVID-19.

Model 3 added a set of control variables associated with the respondents’ demographic and socioeconomic characteristics. Most findings were consistent with those derived from Models 1 and 2, the greater the Internet searching skills, the greater the likelihood of actively seeking COVID-19-related information, as compared with seeking no information. Respondents with a moderate level of disaster mitigation tended to acquire information on COVID-19 more passively than those with a low level of disaster mitigation. A high level of disaster mitigation was also positively associated with being active information-seekers. The results also confirmed that previous experience with flooding was positively associated with being active information seekers, although the association was marginally significant. These findings indicate that flooding experience and preparedness are significantly associated with how individuals are likely to seek health-related information during the COVID-19 pandemic. However, we found no evidence that flooding experience and preparedness function as mediators between the digital divide and COVID-19-related information-seeking behaviors.

A series of multinomial logistic models were estimated that explain engagement in COVID-19 preventive behaviors (Table 6). Model 4 displays the results of the reduced model, which contains only the digital divide-related variables. The estimated coefficients indicated that respondents with a high level of Internet searching skills tended to actively engage in preventive behaviors compared to those with low Internet searching skills.

**Table 6:** Multinomial regression models that explain COVID-19-related information-seeking behaviors of respondents.

Variable		Model 1 (reference = Information non-seekers)				Model 2 (reference = Information non-seekers)				Model 3 (reference = Information non-seekers)			
		Passive information seekers		Active information seekers		Passive information seekers		Active information seekers		Passive information seekers		Active information seekers	
		Coeff.	S.E	Coeff.	S.E	Coeff.	S.E	Coeff.	S.E	Coeff.	S.E	Coeff.	S.E
Digital divide variables	Accessibility to the Internet (ref. no subscription)												
	Through a high-speed internet subscription	0.760	0.914	0.230	0.996	0.676	0.928	−0.198	1.055	0.221	1.109	0.163	1.380
	Through a smartphone data plan	0.054	0.986	−0.326	1.093	−0.143	1.008	−0.414	1.160	−0.378	1.188	1.175	1.505
	Dual subscriptions	1.789^+^	0.948	1.257	1.035	1.609^+^	0.967	1.079	1.100	1.299	1.145	2.014	1.454
	Proficiencies in internet searching skill (ref. Low level)												
	Medium level	−0.230	0.512	1.180	0.812	−0.192	0.525	1.223	0.846	−0.259	0.622	0.904	1.063
	High level	0.275	0.513	2.561^**^	0.806	0.287	0.528	2.313^**^	0.819	0.196	0.637	2.512^*^	1.063
	Proficiencies in social media usage skill (ref. Low level)												
	Medium level	0.589	0.513	−0.475	0.713	0.598	0.524	−0.523	0.764	0.624	0.607	−1.003	0.913
	High level	0.151	0.535	−0.066	0.675	0.051	0.550	−0.012	0.716	−0.078	0.649	0.071	0.855
Flooding-related variables	Experience of flooding (ref. Experienced flooding)												
	Never experienced					−0.123	0.410	−1.246^*^	0.482	0.401	0.471	−1.103^+^	0.588
	Degree in disaster mitigation (ref. Low level of disaster mitigation)												
	Medium level of disaster mitigation					0.621	0.461	−0.673	0.736	1.237^*^	0.563	0.014	0.878
	High level of disaster mitigation					0.203	0.445	1.502^**^	0.533	0.541	0.514	1.667^*^	0.656
Sociodemographic variables		Not Included		Not Included		Not Included		Not Included		Included		Included	
Intercept		−0.909	0.904	−2.301^*^	1.091	−0.922	0.970	−1.824	1.186	−1.556	1.366	−1.951	1.577
N		190.000				187.000				182.000			
Log-likelihood		−183.019				−164.499				−132.472			
Pseudo-R^2^		0.103				0.181				0.325			

*p* + <0.1; p* < 0.05; p** < 0.01; p*** < 0.001.

Model 5 also included flooding-related variables. In contrast to the findings of Model 4, the variables that indicated levels of proficiency in Internet searching become insignificant. Experience with flooding was positively associated with the likelihood of selectively and actively engaging in preventive behaviors. Furthermore, the degree of active disaster mitigation was positively associated with active and selective engagement in preventive behaviors.

Model 6, which includes a set of control variables, indicated that respondents with flooding experience and high levels of disaster mitigation were likely to actively engage in preventive behaviors against COVID-19. These findings were consistent with the results derived from Model 5.

A series of binary logistic regression models were estimated that explained the likelihood of acquiring a particular information source for COVID-19 (see Table 7). These models were designed to explore the conditions under which FVSH residents were likely to rely on a particular information source. Model 7 compares the likelihood of relying on mass media with the likelihood of relying on other information sources. The results indicate that respondents with medium and high levels of engagement in disaster mitigation are likely to use mass media as a primary information source.

**Table 7:** Multinomial regression models that explain the engagement in COVID-19 preventive behaviors.

Variable		Model 4 (reference = Minimal preventive behaviors against COVID-19)				Model 5 (reference = Minimal preventive behaviors against COVID-19)				Model 6 (reference = Minimal behaviors against COVID-19)			
		Passive preventive behaviors		Active preventive behaviors		Passive preventive behaviors		Active preventive behaviors		Passive preventive behaviors		Active preventive behaviors	
		Coeff.	S.E	Coeff.	S.E	Coeff.	S.E	Coeff.	S.E	Coeff.	S.E	Coeff.	S.E
Digital Divide Variables	Accessibility to the internet (ref. No subscription)												
	Through a high-speed internet subscription	−0.301	0.837	0.583	1.229	−0.774	0.964	0.189	1.322	−1.192	1.395	−0.309	1.748
	Through a smartphone data plan	−1.227	0.936	0.185	1.302	−1.750	1.088	−0.073	1.407	−2.562^+^	1.511	−0.081	1.811
	Dual subscriptions	−0.377	0.873	1.594	1.241	−0.820	1.004	1.382	1.337	−1.795	1.434	1.310	1.738
	Proficiencies in internet searching skill (ref. Low level)												
	Medium level	1.009^+^	0.531	−0.716	0.682	1.185^*^	0.598	−0.617	0.728	1.418^+^	0.747	−0.865	0.893
	High level	1.312^*^	0.555	1.112^*^	0.566	1.190^+^	0.628	0.918	0.623	0.868	0.770	0.994	0.766
	Proficiencies in social media usage skill (ref. Low level)												
	Medium level	−0.609	0.528	0.653	0.645	−0.506	0.586	0.563	0.692	−0.346	0.696	0.647	0.813
	High level	−0.350	0.543	0.986	0.662	−0.479	0.616	0.749	0.716	−0.218	0.752	0.897	0.819
Flooding variables	Experience of flooding (ref. Experienced flooding)												
	Never experienced					−1.613^**^	0.474	−1.838^***^	0.488	−1.594^**^	0.562	−1.851^**^	0.574
	Degree in disaster mitigation (ref. Minimal preventive behaviors in disaster mitigation)												
	Passive mitigation behavior					1.335^*^	0.528	0.345	0.580	1.465^*^	0.623	0.369	0.693
	Active mitigation behavior					1.887^***^	0.514	1.799^***^	0.516	2.275^***^	0.625	1.792^**^	0.603
Sociodemographic variables		Not Included		Not Included		Not Included		Not Included		Included		Included	
Intercept		−0.261	0.830	−2.247	1.256	0.303	1.015	−1.228	1.380	−1.334	1.639	−1.441	1.922
N		190.000				187.000				182.000			
Log-likelihood		−186.117				−163.090				−132.899			
Pseudo-R^2^		0.105				0.203				0.333			

*p* + <0.1; p* < 0.05; p** < 0.01; p*** < 0.001.

Model 8 compares the likelihood of using online information with that of relying on other sources. Unlike Model 7, the estimated coefficients indicate that respondents who engaged in disaster mitigation either passively or actively tended to use online information more than any other sources, compared with those who only marginally engaged in disaster mitigation.

Model 9 compares the likelihood of respondents relying on governments or experts with that of relying on other sources. Interestingly, respondents with access to high-speed Internet, smartphone data plans, or both were more likely to use alternative sources to research COVID-19-related information, rather than relying on government-provided information or experts. Statistically significant associations were found with Internet searching skills and increased reliance on official government-provided information. Respondents with medium or high-level Internet skills tended to rely on government-provided information or experts more than any other source. As respondents actively engaged in disaster mitigation, they tended to obtain increasingly more COVID-19-related information from governments or experts—although, at the time of the survey in March 2021, there was ongoing controversy and inconsistency between the information provided by the state government and the CDC.

Model 10 compares respondents who rely on personal networks (e.g., property managers and families) as an information source to those who rely more on other sources (e.g., mass media, online information, governments, or experts). The results indicate that those using both forms of Internet subscription (i.e., high-speed Internet and smartphone data plan) tended to rely more on personal networks than other channels. Furthermore, residents who engaged in active disaster mitigation were more likely to use personal networks than other sources of information.

Model 11 compares respondents who use multiple information sources with those who use a single information source. The results reveal that a high degree of Internet search skills was positively associated with the likelihood of using multiple sources rather than a single source when acquiring COVID-19-related information. Moreover, a medium-to-high level of disaster mitigation was also associated with the use of multiple information sources.

Across all the estimated models, the variables that reflect the degree of disaster mitigation recorded statistically significant results. This consistent pattern suggests that FVSH residents with a high degree of flood risk preparedness are significantly more likely to explore COVID-19 risk information through various channels. However, we found no evidence to support proficiency in social media usage being associated with the increased diversity of channels used when researching COVID-19 information.

## 4 Discussion

This study explored the associations between digital capabilities, past flooding experience, preparedness for flooding, and information-seeking and preventive behaviors in response to COVID-19 among FVSH residents in Florida. The results have revealed several noteworthy findings. First, information-seeking and preventive behaviors in response to COVID-19 are closely associated with proficiency in Internet searching, but not with overall access to the Internet or social media usage skills. Specifically, Internet-savvy FVSH residents are more likely to actively seek COVID-19-related information and engage in preventive actions in response to situations relating to COVID-19. Moreover, given that many emergency management agencies rely on online and social media platforms to provide up-to-date and engaged communication during disasters, the capability to access such platforms may be essential for encouraging FVSH residents to take such desired actions. These findings are consistent with the results of previous studies which have demonstrated that compared to conventional mass media, online information channels, such as official agencies’ websites or online dashboards that display real-time risk information, can do a better job of providing relevant information about disasters and appropriate preventive measures (41). The statistically insignificant outcomes of social media-related variables may be due to the potentially mixed effects of social media posts on responses to COVID-19. Social media posts contain not only verified information but also distorted information, with filtering unreliable information having become a near impossibility in the social media age (42). Unfortunately, many users trust misinformation, and fewer can deny or double such claims based on proper reasoning (43). Thus, if social media skills exposed respondents to both reliable and unreliable information during this period, their associations with information-seeking and preventive behaviors may be mixed, resulting in insignificant results being obtained. This potential explanation may be compatible with the relatively smaller estimated coefficients for medium or high levels of social media usage skills shown in Tables 6, 7.

Second, the results confirmed that variables indicating past flooding experiences and preparedness for floods were significantly associated with COVID-19 responses. Specifically, FVSH residents who had previously experienced flooding and indicated a high degree of preparedness for flooding were more likely to actively seek COVID-19-related health information and actively engage in prevention behaviors. These findings suggest that in multi-hazard environments, experience with and preparedness for a particular type of non-pandemic disaster may foster proactive information-seeking and preventive behaviors, supporting the theory that experiences and responses to multiple types of disasters are interdependent (44). Moreover, respondents with a high level of flood mitigation tended to use more diverse channels to search for COVID-19 risk information. This finding suggests that FVSH residents who are sufficiently prepared for flooding tend to acquire COVID-19-related information more comprehensively and actively. This finding may also imply that enhancing preparedness for non-pandemic disasters may help individuals be well-prepared for potential future pandemics. This pattern could be relevant in the context of an infodemic, where individuals are faced with an overwhelming amount of information—some of it unreliable. It is plausible that individuals accustomed to navigating complex risk environments, such as those involving floods, develop stronger risk awareness and information discernment skills. These skills may, in turn, enable them to more effectively evaluate, filter, and integrate health information during a pandemic. Thus, strengthening disaster preparedness may have the added benefit of enhancing individual resilience against the harmful effects of infodemics. However, it is important to note that the study found no evidence supporting the idea that past flooding experiences and degree of preparedness mediate the relationship between digital divides and COVID-19-related responses (Table 8).

**Table 8:** Binary logistic models that explain the acquisition of COVID-19 information sources.

Variable		Model 7		Model 8		Model 9		Model 10		Model 11	
		Mass media vs. Others (DV = 0)		Online information (DV = 1) vs. Others (DV = 0)		Government or experts (DV = 1) vs. Others (DV = 0)		Personal networks (DV = 1) vs. Others (DV = 0)		More than two sources vs. Others (DV = 0)	
		Coeff.	S.E	Coeff.	S.E	Coeff.	S.E	Coeff.	S.E	Coeff.	S.E
Digital Divide Variables	Accessibility to the Internet (ref. No subscription)										
	Through a high-speed internet subscription	0.894	1.001	−0.177	1.014	−2.779^*^	1.251	1.371	0.968	−0.008	1.002
	Through a smartphone data plan	1.070	1.070	−0.243	1.078	−3.273^*^	1.320	1.603	1.032	−0.166	1.057
	Dual subscriptions	1.474	1.036	0.983	1.067	−2.115^+^	1.261	2.257^*^	1.003	1.309	1.079
	Proficiencies in internet searching skill (ref. Low level)										
	Medium level	−0.318	0.568	−0.619	0.678	1.389^*^	0.590	0.026	0.562	0.410	0.630
	High level	0.189	0.560	−0.251	0.719	1.381^*^	0.595	0.410	0.545	1.139^+^	0.646
	Proficiencies in social media usage skill (ref. Low level)										
	Medium level	0.510	0.534	−0.082	0.615	0.037	0.562	−0.415	0.527	−0.825	0.610
	High level	0.742	0.574	0.031	0.663	0.110	0.600	−0.169	0.559	−0.470	0.651
Flooding variables	Experience of flooding (ref. Experienced flooding)										
	Never experienced	0.094	0.397	−0.470	0.494	0.675	0.419	−0.203	0.407	0.583	0.480
	Degree in disaster mitigation (ref. Minimal preventive behaviors in disaster mitigation)										
	Passive mitigation behavior	1.444^**^	0.511	1.528^*^	0.698	−0.369	0.506	0.611	0.481	1.804^**^	0.679
	Active mitigation behavior	1.109^*^	0.430	0.916^+^	0.490	1.036^*^	0.451	1.182^**^	0.427	1.209^*^	0.494
Sociodemographic variables		Included		Included		Included		Included		Included	
Intercept		−0.807	1.148	0.178	1.205	−0.692	1.431	−1.334	1.131	−0.492	1.208
N		177.000		181.000		181.000		183.000		177.000	
Log-likelihood		−99.781		−77.255		−94.955		−102.038		−76.213	
Pseudo-R^2^		0.180		0.202		0.231		0.162		0.205	

*p* + <0.1; p* < 0.05; p** < 0.01; p*** < 0.001.

After controlling for flood-related variables, Internet search proficiency was significantly associated with responses to COVID-19, although social media skills remained insignificant across all estimated models. One possible explanation is that proficiency in using social media does not necessarily translate to the ability to critically evaluate the credibility or accuracy of health-related content encountered on these platforms. Prior research has shown that users often struggle to assess the reliability of health information shared on social media, especially when it is embedded in personal narratives or influenced by peer dynamics (45, 46). Unlike general Internet search, which typically involves deliberate information-seeking behavior—often through search engines and curated websites—social media usage may be more passive and shaped by algorithmic exposure to user-generated content (47). As a result, individuals with higher social media skills might not be better equipped to discern misinformation from credible sources, which could limit the impact of such skills on health-related behavioral responses. Additionally, social media environments are often echo chambers where information is filtered through personal networks, potentially reinforcing pre-existing beliefs rather than facilitating informed decision-making (48). These findings highlight an important implication for public health communication: improving digital literacy alone may be insufficient unless efforts specifically address critical evaluation and information discernment skills, particularly within the context of social media platforms.

This study provides evidence supporting the warning and response model (40) highlighting that disaster-related experiences and subsequent readiness may assist individuals in identifying and preparing for future disaster threats. Previous research has provided indirect evidence of the importance of disaster experience in the context of disaster preparedness. For example, individuals who reside in areas frequently threatened by natural disasters may be more likely to recognize the threat (40), take more preventive measures (49), and heed warnings (50) than those who live in areas rarely threatened by disaster. Disaster survivors who have experienced greater levels of property loss and psychological distress may be more attentive to news reports and have a better understanding of the destructive consequences of disasters than survivors who have experienced lower levels of property loss and distress, or those who have not experienced a disaster (38). Our results suggest that this warning and response model can be applied to the relationship between floods and COVID-19; specifically, flooding-related experiences and subsequent readiness are significantly and positively associated with information-seeking and preventive behaviors.

## 5 Conclusion

This study has several limitations. First, as it specifically focused on FVSH by applying a strict screening question, this study had a relatively limited sample size of 200 households. While the screened samples used in the analyses had balanced distributions across sociodemographic groups among FVSH residents and revealed many significant findings, our findings are limited in generalizability and there should be further studies based on the broader population. Second, we used Qualtric to distribute the survey link to qualified participants because of the operating restrictions imposed by the COVID-19 pandemic. This approach inherently required a certain level of Internet access and digital literacy, which may have introduced selection bias by excluding FVSH residents who lacked adequate connectivity or technological skills. As a result, our analysis may have underestimated the role of fundamental aspects of digital access, such as device availability or basic connectivity, which are often more prevalent barriers among vulnerable populations. To address these limitations, future research should consider mixed-mode survey strategies (e.g., telephone or paper-based surveys) or community-based recruitment methods to better capture the experiences of digitally marginalized groups. Third, participants’ digital skills were assessed through self-evaluation, which may be subjective and prone to response bias, as individuals often overestimate or underestimate their actual proficiency depending on confidence or familiarity with technology (51). Although direct observation or performance-based tasks can offer more objective measures (24), such methods are less feasible in studies involving geographically dispersed participants, such as residents of multiple subsidized housing units. Nevertheless, future research could incorporate hybrid approaches, such as short in-survey skill tests or scenario-based assessments, to improve the validity of digital skill measurement while maintaining practical feasibility. Finally, due to the cross-sectional survey design, our results revealed associations between many attributes, but further investigations are needed to identify causal relationships. In particular, future research should consider longitudinal or experimental designs to examine how the timing and content of risk communication influence behavioral responses over time, especially in relation to the timing of COVID-19 infection and exposure to public health information.

This study explores the relatively under-examined associations between individuals’ digital capabilities, experience with and preparedness for flooding, and responses to COVID-19 based on survey data collected from subsidized residents living in flood-vulnerable communities in Florida, USA during the COVID-19 period. Our findings have the following actionable implications for minimizing multiple layers of vulnerability in subsidized housing neighborhoods. First, local public agencies for disaster mitigation should expand their programs to provide individuals with training in digital skills and online information seeking. Given that previous studies have also highlighted that digital skills tend to lead individuals to take preventive actions to mitigate disaster risks, digital literacy training should evidently be integrated into risk awareness campaigns. For instance, Philadelphia’s Digital Literacy Alliance funds community-based programs that promote digital inclusion through peer leadership and volunteer engagement. Austin’s “Unlocking the Connection” initiative, in partnership with Google Fiber, provides low-cost internet, training, and devices to public housing residents. Minneapolis and Hennepin County similarly offer free Wi-Fi access, digital skills workshops, and digital navigator services to underserved populations. These programs highlight key implementation strategies such as public-private partnerships, community outreach, equitable access to devices and connectivity, and training focused on both technical skills and critical online information evaluation. By adopting similar approaches, local governments can effectively integrate digital literacy into risk awareness and disaster mitigation efforts. Second, to maximize the contributive role of social media in disseminating risk information and promoting risk mitigation actions, there should be consideration of how social media users are exposed to the posts. More reliable information from the social media accounts of federal/state/county/local government agencies (first responders) could be more actively promoted for instance. Third, this study presents a holistic strategy for enhancing overall preparation in the context of multiple disaster risks. Although further studies should be conducted on how preparedness for a particular disaster influences responses to other types of disaster, as this study demonstrates that the abilities to respond to disastrous environments are interconnected, mitigation policies should be jointly designed and implemented across multiple policy domains to improve the resilience of individuals exposed to multi-hazard environments.

## Data Availability

The datasets presented in this article are not readily available because the data that has been used is confidential.

## Footnotes

^1^Centers for Disease Control and Prevention. COVID Data Tracker (2025). Available online at: https://covid.cdc.gov/covid-data-tracker/#trends_totaldeaths_select_00 (Accessed April 16, 2025).

^2^Given our focus on subsidized housing in flood-prone areas, respondents are likely to be resided in urban rather than rural areas. This pattern is largely explained by Florida’s geography and historical housing development practices. Many of Florida’s major urban centers—such as Miami, Tampa, Jacksonville, and Fort Lauderdale—are located along the coastline and in low-lying zones, making them inherently more vulnerable to flooding. These urban centers are home to a greater number of the state’s population, which includes low-income households that rely on government-supported housing (52). Moreover, subsidized housing developments have historically been concentrated in regions with lower land values, frequently situating them in environmentally vulnerable urban areas [PreventionWeb. Why affordable housing is more vulnerable to disasters (2023). Available online at: https://www.preventionweb.net/drr-community-voices/why-affordable-housing-more-vulnerable-disasters (Accessed April 16, 2025)]. Consequently, the co-location of flood-prone zones and subsidized housing does not substantially alter the urban–rural balance; rather, it reinforces the current trend of urban concentration.
